# Novel six-month all oral treatment of pre-extensively drug-resistant tuberculosis in Canada: New treatment options present new implementation challenges

**DOI:** 10.14745/ccdr.v49i01a04

**Published:** 2023-01-05

**Authors:** William Connors, Cesilia Nishi, Inna Sekirov, Victoria Cook, James Johnston

**Affiliations:** 1 Department of Medicine, University of British Columbia, Vancouver, BC; 2 Tuberculosis Services, BC Centre for Disease Control, Vancouver, BC; 3 Division of Infectious Diseases, Vancouver General Hospital, Vancouver, BC; 4 Department of Pharmaceutical Sciences, Vancouver General Hospital, Vancouver, BC; 5 Faculty of Pharmaceutical Sciences, the University of British Columbia, Vancouver, BC; 6 BCCDC Public Health Laboratory, BC Centre for Disease Control, Vancouver, BC; 7 Pathology and Laboratory Medicine, University of British Columbia, Vancouver, BC

**Keywords:** tuberculosis, pretomanid, bedaquiline, linezolid, extensively drug-resistant tuberculosis, multi-drug-resistant tuberculosis, Canada

## Abstract

Drug-resistant tuberculosis (TB) is a major global health challenge in part because there are fewer effective treatments and these treatments have been prolonged and more toxic. The evidence base for more effective, shorter, standardized treatments is evolving rapidly. Herein, we report the first case of pre-extensively drug-resistant pulmonary TB treated with a novel six-month all oral bedaquiline, pretomanid and linezolid (BPaL) regimen in Canada. Recent clinical trial data supporting BPaL therapy is presented in the context of current and evolving clinical guidelines. In this article, we highlight significant implementation challenges and make recommendations for what needs to be addressed to ensure safe programmatic use of BPaL in Canada. Key recommendations include the development of infrastructure for timely access to novel TB drug susceptibility testing, streamlining access to novel TB drugs, and cautious use of such drugs in collaboration with care teams with expertise in drug-resistant TB management.

## Introduction

Drug-resistant tuberculosis (TB) is associated with disproportionate morbidity and mortality related to prolonged, more toxic, and less effective treatments. In 2020, the World Health Organization (WHO) estimated 10 million people developed TB disease, including 500,000 people with rifampin-resistant/multi-drug-resistant TB (RR/MDR) ((1)). In recent years, a series of clinical trials evaluating novel (bedaquiline, pretomanid) and repurposed (linezolid, clofazimine, fluoroquinolones) oral anti-TB drugs have demonstrated the potential for shorter, less toxic and highly effective treatment of drug-resistant forms of TB ((2–5)). Informed by accumulating clinical evidence, the WHO recently released updated clinical guidance endorsing the use of a novel all oral short course (6–9 month) regimen of bedaquiline, pretomanid and linezolid (BPaL) for RR/MDR and pre-extensively drug-resistant (pre-XDR) TB (**Table 1**) ((6)). While this represents a major advancement in TB treatment, it also presents important operational challenges, including timely access to resistance testing and drug procurement. We present our experience treating a person with pre-XDR TB with BPaL—the first patient to complete this regimen in Canada—and highlight key implementation challenges.

**Table 1:** Updated World Health Organization drug-resistant tuberculosis definitions, 2021^a^

Type of drug resistance	Definition
Mono/poly-drug-resistant	Single or multi-drug resistance not meeting MDR/XDR criteria
MDR^b^	Concurrent rifampin and isoniazid resistance
Pre-XDR	MDR criteria plus fluoroquinolone resistance^c^
XDR	Pre-XDR criteria plus bedaquiline or linezolid resistance

Abbreviations: MDR, multi-drug-resistant; pre-XDR pre-extensively drug-resistant; XDR, extensively drug-resistant

^a^ Taken from reference (7)

^b^ WHO classifies both rifampin-resistant (RR) and MDR TB as clinically similar in management and RR/MDR is combined in global surveillance data

^c^ Either levofloxacin or moxifloxacin

## Case report

**Figure 1:**
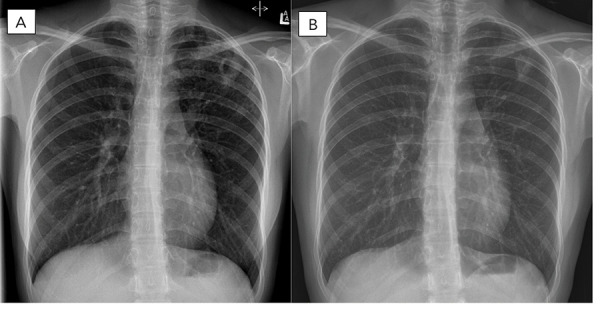
Chest X-ray (posterior-anterior film)^a^ ^a^ The left X-ray showing left upper lung thick walled cavitary lesion pretreatment with the right X-ray showing improvement on follow-up six-month post completion of treatment

In March 2021, an 18-year-old female was referred to a provincial TB program with a five-month history of cough and several weeks of night sweats. Chest radiograph revealed left upper lobe cavitation (**Figure 1**). Sputum samples demonstrated acid-fast bacilli on smear, which was confirmed as *Mycobacterium tuberculosis* complex by polymerase chain reaction assay targeting the IS6110 and *mpt*64 genes. The patient was born in China and had moved to Canada three years prior to her TB diagnosis. She had no known prior TB exposure or treatment, was a non-smoker and was on no medications. Baseline investigations were negative for human immunodeficiency virus, hepatitis B/C and diabetes.

A week following referral, she was initiated on standard first-line, four-drug anti-TB therapy, and continued home isolation. Three weeks later, first-line drug phenotypic susceptibility testing indicated the possibility of multi-drug resistance, further confirmed by both genotypic and repeat phenotypic testing (**Table 2**). The isolate was sent to the National Reference Centre for Mycobacteriology (Winnipeg, Manitoba) and the National Jewish Hospital Laboratory (Denver, Colorado) for second-line drug testing. The patient was admitted to the provincial hospital-based TB Unit to establish therapy geared towards pre-XDR TB and continue isolation. Three months post-diagnosis, two months into hospitalization, phenotypic second-line drug susceptibility results were finalized by the National Reference Centre for Mycobacteriology (Table 2), but phenotypic drug susceptibility testing for bedaquiline and pretomanid were not available in any of the North American labs we contacted.

**Table 2:** Sputum mycobacterial culture drug susceptibility test results

Drug	MIC (mg/L)	Interpretation	Testing method	Testing laboratory
Rifampin	At least 1	Resistant	MGIT	Provincial
Isoniazid	At least 0.4	Resistant	MGIT	Provincial
Pyrazinamide	At least 100	Resistant	MGIT	Provincial
Ethambutol	At least 5	Resistant	MGIT	Provincial
Moxifloxacin	At least 0.25	Resistant	MGIT	Provincial
Rifabutin	At least 0.5	Resistant	MGIT	National
Amikacin^a^	At least 0.1	Resistant	MGIT	National
Ethionamide	At least 5	Resistant	MGIT	National
Linezolid^b^	1	Susceptible	MGIT	National
*para*-Aminosalicylic acid	4	Susceptible	MGIT	National
Clofazimine	0.12 or less	Tentative susceptible	Agar dilution	US
Cycloserine	60	Susceptible	Agar dilution	US

Abbreviations: MGIT, Mycobacteria Growth Indicator Tube; US, United States

^a^ Also resistant to kanamycin, capreomycin and streptomycin

^b^ Extended incubation was required; result did not conform to the standard protocol recommended by Clinical and Laboratory Standards Institute

**Figure 2:**
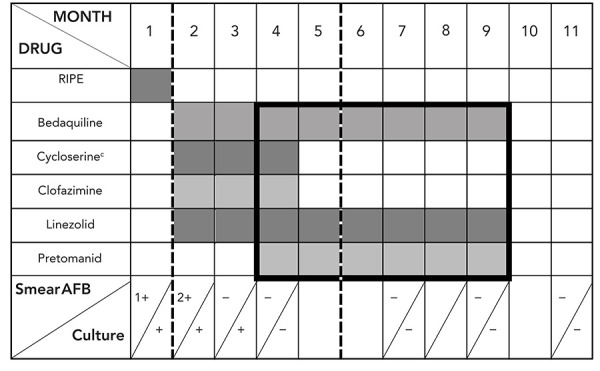
Treatment timeline and details^a,b^ Abbreviations: AFB, acid-fast bacilli; RIPE, rifampin, isoniazid, pyrazinamide, ethambutol at standard weight-based dosing ^a^ Drug dosing (weight: 45 kg): bedaquiline 400 mg PO OD x 2 weeks then 200 mg 3 times per week, cycloserine 250 mg PO BID, linezolid 1,200 mg PO OD month 2 then 600 mg PO OD months 3 to 9, clofazimine 100 mg PO OD, pretomanid 200 mg PO OD ^b^ Dark line/box: BPaL (bedaquiline pretomanid, linezolid), dashed line; hospital admission and discharge ^c^ Together with pyridoxine (B6) 100 mg

Treatment is summarized in **Figure 2**. In hospital, treatment was initially modified to four presumed effective medications (bedaquiline, clofazimine, cycloserine, linezolid) once Health Canada Special Access Program (SAP) approval and medication procurement completed. Based on recent reports of effective use of combination therapy with pretomanid ((3)), SAP approval was sought from Health Canada. While SAP approval for use of pretomanid was obtained quickly, it took three weeks from approval to initiate therapy due to manufacturer delays related to establishing an account to order and purchase the drug. Once the patient was established on pretomanid, a consensus decision among TB Unit physicians was made to stop clofazimine and cycloserine and proceed with a BPaL six-month regimen adhering to operational research conditions outlined by WHO ((8)). This decision was informed by the limited extent of TB disease and desire to limit toxicity and social disruption in this young, school-aged patient. The patient provided informed consent to proceed with treatment. Given her small body habitus (45 kg) and concern about high rates of linezolid-associated adverse events at 1,200 mg daily dosing used in the published BPaL regimen ((3)), the dose was reduced to 600 mg daily after one month informed by published pharmacokinetic parameters. Peak and through linezolid serum drug levels were performed via Infectious Disease Pharmacokinetics Laboratory (Gainesville, Florida) confirming adequate drug exposure at 600 mg daily ((9)).

**Figure 3:**
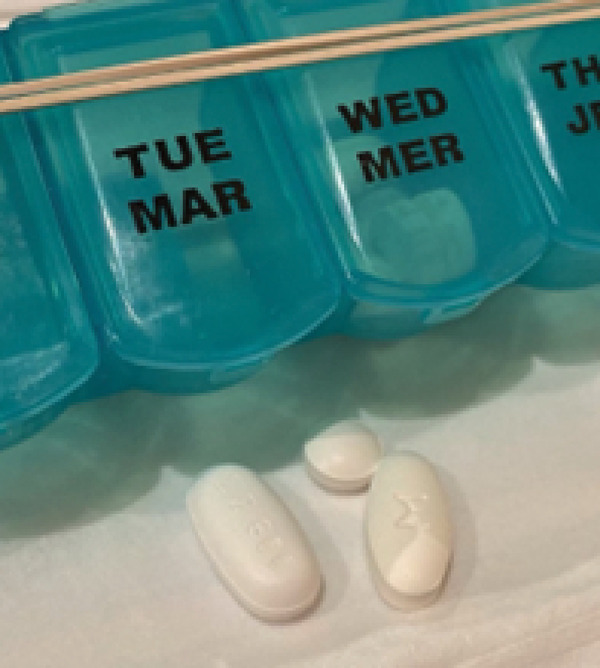
Photo of daily BPaL medications^a^ ^a^ Medication submitted along with video of pill ingestion by the patient as asynchronously daily video-assisted directly observed therapy

Unable to home isolate, the patient remained hospitalized until sputum culture was confirmed negative (month 5) (**Figure 3**). Outpatient treatment was via daily observed dosing either in-person or via asynchronous video-assisted directly observed therapy (Figure 3). Monthly blood work, including complete blood counts, kidney function, liver enzymes and lipase levels, along with electrocardiogram, demonstrated no adverse treatment effects that required modification. During the third month of treatment, the patient developed a non-progressive multifocal pruritic papular rash without systemic symptoms that responded symptomatically to topical corticosteroids and resolved with cessation of clofazimine and cycloserine.

One month after hospitalization and treatment optimization, all TB symptoms had resolved and sputum smears and cultures were consistently negative. Serial follow-up chest imaging demonstrated improvement. In November 2021, BPaL regimen treatment was stopped. More than six months post treatment the patient remained symptom free with negative sputum cultures and improved chest imaging (Figure 2 and Figure 3).

## Discussion

Canadian guidelines currently recommend MDR/pre-XDR TB treatment with individualized all oral regimens consisting of at least four effective medications for at least 20 months ((10,11)). However, the evidence base for shorter standardized treatment, such as BPaL, has evolved rapidly since the publication of the guidelines ((6)). This case illustrates the safe and effective use of the novel BPaL regimen to treat pre-XDR TB in Canada. It also highlights key implementation challenges: timely and accessible TB drug susceptibility testing; medication access; and program-level knowledge about appropriate use.

The evidence base for BPaL comes from three recent clinical trials. The open label, single group Nix-TB trial published in 2020, first evaluated six-month BPaL treatment of MDR/pre-XDR pulmonary TB and demonstrated a remarkable 90% of participants experienced favourable outcomes. This is in dramatic contrast to the consistently fewer than 70% favourable outcomes with prior guideline-based regimens. However, participants in Nix-TB experienced significant toxicity; with a linezolid dose of 1,200 mg daily, more than 80% of participants reported adverse effects, and 71% required linezolid treatment interruption ((3)). To validate these findings and evaluate toxicity mitigation strategies, the multinational phase II/III TB-PRACTECAL trial compared six-month BPaL using variable linezolid dosing (600 mg daily for four months then 300 mg or 600 mg three times per week for two months) with or without moxifloxacin or clofazimine, against locally accepted standard of care ((12)). While the multinational, partially blinded phase III ZeNIX trial compared six-month BPaL using variable linezolid dose (1,200 mg or 600 mg) and duration (two or six months) ((4,13)). Results from these trials demonstrate that more than 80% of participants experienced successful treatment outcomes with improved tolerability at reduced linezolid doses (600 mg daily); however, final results of the TB-PRACTECAL trial have not yet been published ((4,12)).

Based on these findings, BPaL regimens were recently endorsed by the WHO as potential treatment options for highly drug-resistant TB ((6)). To preserve effectiveness and ensure safety, careful patient selection and treatment oversight must be central to programmatic use. To this end, the WHO outlined potential BPaL patient eligibility criteria in their 2020 consolidated guidelines on drug-resistant tuberculosis treatment ((8)). The central criteria for patient selection are as follows: informed consent; side effect monitoring; and TB susceptibility to medications used. It should also be underscored that there is currently a lack of evidence for use of BPaL in children (younger than 15 years of age) and for extra-pulmonary TB. For Canadian clinicians and pharmacists who may have limited familiarity with the novel drugs in BPaL, the recently updated Canadian Tuberculosis Standards 8^th^ Edition provides a useful overview of these medications ((9)).

Despite increasing prevalence of resistance—including to BPaL drugs—and recommendations that susceptibility to treatment drugs be laboratory-confirmed in all people with TB, the ability to perform drug susceptibility testing for novel TB drugs, like pretomanid and bedaquiline, remains limited globally and currently unavailable in Canada ((6,8,15)). Clinical and Laboratory Standards Institute guidelines—a common set of laboratory standards used worldwide—do not provide recommendations for test performance or interpretation to either of these drugs. While breakpoints for susceptibility testing to bedaquiline and delamanid (a nitroimidazooxazine class drug like pretomanid) are available through European Committee on Antimicrobial Susceptibility Testing guidelines, shortages of drug available for test set up (in the case of delamanid) and lack of readily available well-characterized organisms for validation and standardization impede implementation. These factors contributed to lack of bedaquiline and pretomanid susceptibility testing for our case. Even when drug susceptibility testing is available for a drug, delayed results impact timely decision-making and clinical utility. In our case it took three months to obtain finalized linezolid susceptibility results due to specimen shipment delays, testing capacity limitations, and isolate specific incubation/growth issues. As such, expanded treatment regimens should be considered while awaiting susceptibility testing results and BPaL regimens may not be appropriate if there is concern for constituent drug resistance and susceptibility testing unavailability. Borrowing from non-BPaL treatment recommendations, additional medications such as moxifloxacin, clofazimine and cycloserine could be included while awaiting susceptibility testing results ((6,10)).

## Recommendations

Safe and effective use of BPaL for treatment of persons with drug-resistant TB in Canada presents multiple significant implementation challenges. To address these challenges, we recommend the following:

• Prioritizing validated and accessible preferred drug-resistant TB drug (bedaquiline, pretomanid, clofazimine, linezolid, cycloserine) susceptibility testing via collaboration between regional labs and national and international reference and research centres

• Streamlining access to novel TB medicines by using shared, standardized eligibility criteria as well as harmonized priority application and procurement pathways with Health Canada SAP and drug suppliers

• Limiting this regimen to operational research conditions that reflect eligibility criteria and implementation guidelines similar to those outlined by the WHO ((8)), particularly in the absence of documented drug susceptibility to BPaL drugs (specifically the now more widely used linezolid and bedaquiline)

Alongside these needed system improvements, in accordance with national and international best practice guidelines, case-by-case management of people with drug-resistant TB should occur in close collaboration with a team of experienced physicians, nurses and pharmacists ((7,10,11)).

## Conclusion

New treatment options for drug-resistant TB represent a major advancement for global TB elimination efforts. However, system improvements and continued close operational oversight will be required for sustainable effective integration of these new treatments into TB care in Canada.
